# Programmatic assessment of competency-based workplace learning: when theory meets practice

**DOI:** 10.1186/1472-6920-13-123

**Published:** 2013-09-11

**Authors:** Harold GJ Bok, Pim W Teunissen, Robert P Favier, Nancy J Rietbroek, Lars FH Theyse, Harold Brommer, Jan CM Haarhuis, Peter van Beukelen, Cees PM van der Vleuten, Debbie ADC Jaarsma

**Affiliations:** 1 Quality Improvement in Veterinary Education, Faculty of Veterinary Medicine, Utrecht University, Utrecht, The Netherlands; 2 Department of Educational Development and Research, Faculty of Health, Medicine, and Life Sciences, Maastricht University, Maastricht, The Netherlands; 3 Department of Obstetrics and Gynaecology, VU University Medical Centre, Amsterdam, The Netherlands; 4 Department of Clinical Sciences of Companion Animals, Faculty of Veterinary Medicine, Utrecht University, Utrecht, The Netherlands; 5 Department of Equine Sciences, Faculty of Veterinary Medicine, Utrecht University, Utrecht, the Netherlands; 6 Evidence-Based Education, Academic Medical Centre, University of Amsterdam, Amsterdam, The Netherlands

**Keywords:** Programmatic assessment, Workplace learning, Undergraduate (veterinary) medical education, (Peer) Feedback, Mentoring, Personal development

## Abstract

**Background:**

In competency-based medical education emphasis has shifted towards outcomes, capabilities, and learner-centeredness. Together with a focus on sustained evidence of professional competence this calls for new methods of teaching and assessment. Recently, medical educators advocated the use of a holistic, programmatic approach towards assessment. Besides maximum facilitation of learning it should improve the validity and reliability of measurements and documentation of competence development. We explored how, in a competency-based curriculum, current theories on programmatic assessment interacted with educational practice.

**Methods:**

In a development study including evaluation, we investigated the implementation of a theory-based programme of assessment. Between April 2011 and May 2012 quantitative evaluation data were collected and used to guide group interviews that explored the experiences of students and clinical supervisors with the assessment programme. We coded the transcripts and emerging topics were organised into a list of lessons learned.

**Results:**

The programme mainly focuses on the integration of learning and assessment by motivating and supporting students to seek and accumulate feedback. The assessment instruments were aligned to cover predefined competencies to enable aggregation of information in a structured and meaningful way. Assessments that were designed as formative learning experiences were increasingly perceived as summative by students. Peer feedback was experienced as a valuable method for formative feedback. Social interaction and external guidance seemed to be of crucial importance to scaffold self-directed learning. Aggregating data from individual assessments into a holistic portfolio judgement required expertise and extensive training and supervision of judges.

**Conclusions:**

A programme of assessment with low-stakes assessments providing simultaneously formative feedback and input for summative decisions proved not easy to implement. Careful preparation and guidance of the implementation process was crucial. Assessment for learning requires meaningful feedback with each assessment. Special attention should be paid to the quality of feedback at individual assessment moments. Comprehensive attention for faculty development and training for students is essential for the successful implementation of an assessment programme.

## Background

In recent decades, society and professional associations have come to place increasing importance on generic competencies and evidence of sustained professional competence [1,2], giving rise to competency-based education with emphasis on outcomes, competencies, and learner-centeredness [3]. The shift to competency-based education challenged medical educators to develop new methods of teaching and assessing clinical competence. Based on the notion that using one single assessment method can compromise the reliability, validity, impact on learning, and other quality criteria of assessment [4], Van der Vleuten and Schuwirth proposed a holistic, programmatic approach to assessment aimed at improving the validity and reliability of measurements and documentation of competency development [5]. In recent years, developments are seen in undergraduate and postgraduate education to design programmes of assessment monitoring trainees’ progression towards defined standards of performance [6-9]. Assuming that combining different assessment instruments and supplementing traditional instruments with modern ones can not only counteract the downsides of using a single assessment instrument [5,10-12] but also provide a holistic overview of students’ competency development for formative feedback and summative decisions [12], Van der Vleuten et al. proposed a model of programmatic assessment aimed at optimising the education and certification functions of assessment [13]. They formulated a set of theoretical principles to meet the requirements of maximum facilitation of learning (assessment *for* learning) and maximum robustness of high-stakes decisions (assessment *of* learning), while also supplying information for the improvement of curricular quality [13].

**Figure 1:**
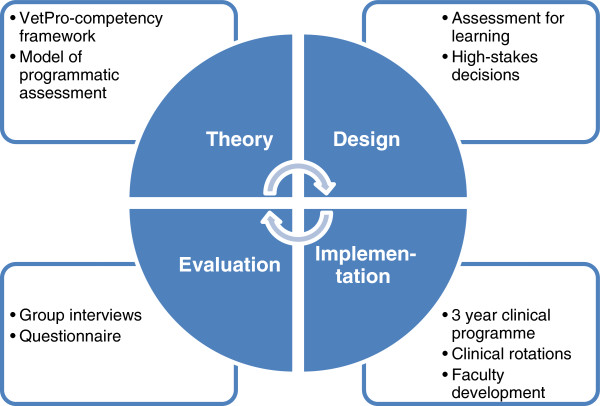
Cycle of design, implementation, evaluation and redesign.

Building on and aiming to advance these theoretical principles, we undertook a development study including evaluation to explore the interaction of theoretical principles with educational practice. The aim of this study was to investigate the nature of learning as it takes place in authentic learning environments, bridging the gap between research and practice. We designed and implemented an assessment programme and collected and analysed quantitative and qualitative evaluation data (Figure 1) to guide redesign. In accordance with the “conventional structure for reporting on experiments that evolve over time” proposed by Collins et al. we consecutively describe the goals and elements of the design and the methods used to collect and analyse the evaluation data [14]. Finally, we present the findings from the analysis of the evaluation data, discussing these in light of the assessment principles informing the programme. Based on the theoretical principles described by Van der Vleuten et al. [13] we identified four overarching challenges to be met by the assessment programme and translated these into research questions:

•Can data from multiple individual assessments be used to combine formative (assessment for learning) and summative (assessment of learning) functions of assessment?

•Can information from individual assessment data points be aggregated meaningfully?

•Can assessment drive desirable learning?

•How can the assessment programme promote reflective and self-directed learning activities?

### The goals and elements of the programme of assessment

#### Setting

A major curriculum reform at the Faculty of Veterinary Medicine, Utrecht University (FVMU) in the Netherlands offered an opportunity to design and test a competency-based assessment programme for the three-year clinical phase of the six-year undergraduate curriculum. Launched in September 2010, the new clinical phase comprises one to seven week clinical rotations in disciplines related to three tracks: equine health, companion animal health, and farm animal health. Students select one track and work side by side with clinical staff in the workplace where they encounter a variety of learning activities. Formal teaching is aimed at promoting in-depth understanding of topics encountered during clinical work.

#### Research team

The research was conducted by a team consisting of clinical supervisors with expertise in curriculum development, assessment, and clinical supervision, faculty with expertise in educational design, and educational researchers with expertise in curriculum development and workplace-based assessment (WBA). Starting their activities in September 2009, the team met in monthly progress meetings, consulting, if necessary, external experts on specific subjects.

#### The design of the assessment programme

The assessment programme was designed in accordance with the model of programmatic assessment proposed by Van der Vleuten et al. [13]. Built around learning activities, assessment activities, supporting activities, intermediate evaluations, and final evaluations, the programme was designed to meet the five main goals formulated by the research team. These goals were based on the theoretical principals and, as a consequence, in alignment with the research questions:

1) To give students insight into their learning and longitudinal competency development.

2) To offer learning opportunities which are also potential assessment opportunities.

3) To ensure that the main focus is on meaningful feedback to further attainment of predefined professional competencies.

4) To promote reflective and self-directed learning activities.

5) To enable faculty to make robust (defensible and transparent) high-stakes (promotion/remediation) decisions.

**Figure 2:**
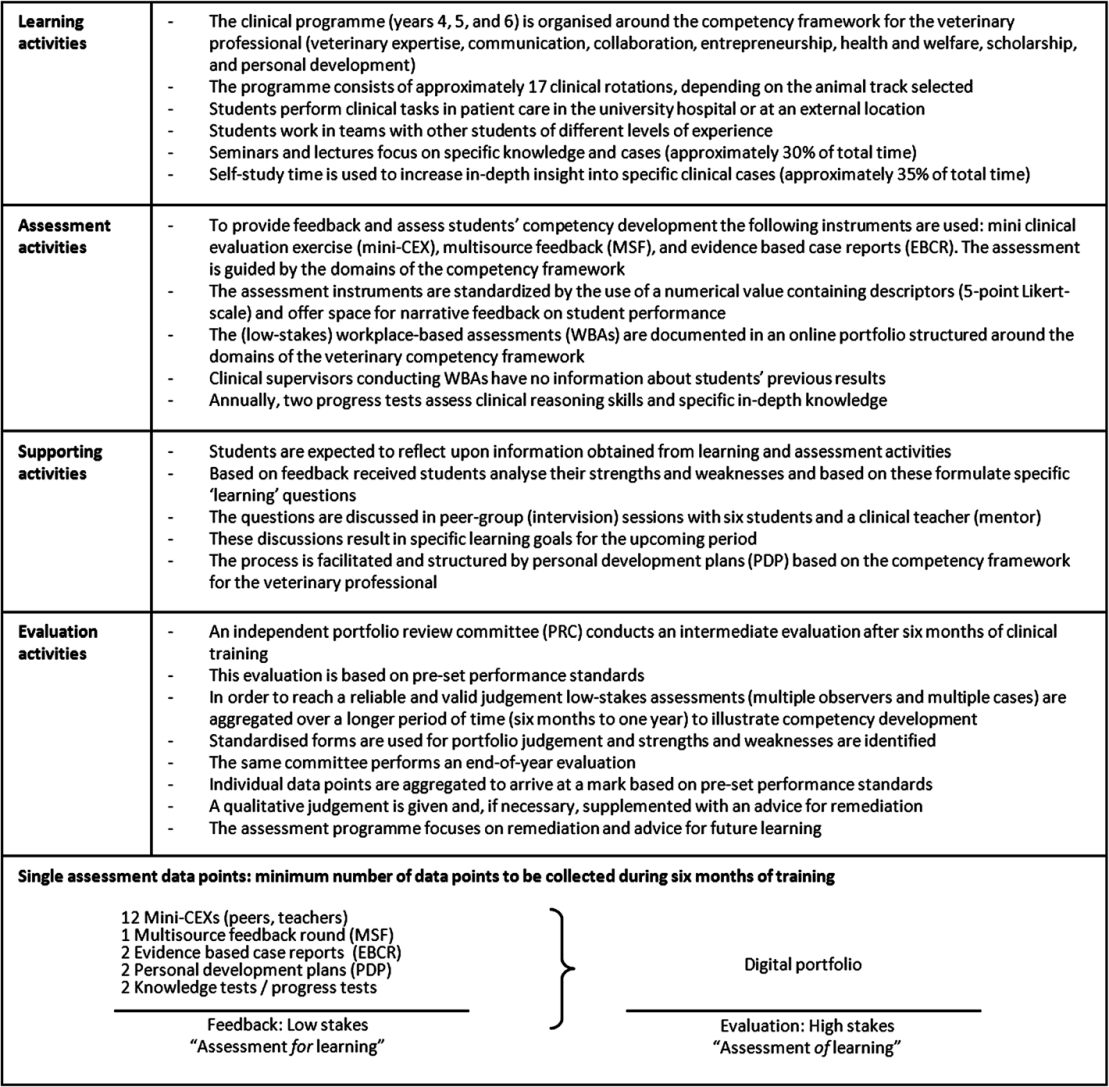
Competency-based assessment programme at FVMU introduced in September 2010.

These starting points and the competency framework for veterinary professionals (VetPro) underpinned the initial assessment blueprint developed by the team [15]. The VetPro competency framework consists of seven domains (Veterinary Expertise, Communication, Collaboration, Entrepreneurship, Health and Welfare, Scholarship, and Personal Development) subdivided in eighteen competencies. The framework was originally developed through a multi-method study with clients and veterinarians representing the full range and diversity of the veterinary profession [15]. The assessment instruments were in alignment with the competency framework to enable aggregation of information in a structured and meaningful way. Several discussion sessions with educational experts and the team resulted in an assessment programme, which, starting in September 2010, was piloted (Figure 2).

The programme focused on the integration of learning and assessment by motivating and supporting students to arrange for WBAs that provide feedback to monitor their competency development. Students were expected to take responsibility for managing and documenting their development. To help students reflect on their learning and assessment activities, supporting activities were offered: small group sessions to discuss learning goals with peers and a clinical supervisor (mentor) and individual student-mentor meetings. Annually, at a six-month interval, an intermediate and a final evaluation was conducted based on predefined performance standards. The primary objective of the intermediate evaluation was to provide students feedback on longitudinal competency development to be used to formulate new learning goals to prepare for the final (high-stakes) evaluation leading to a summative decision (go/no go). Prior to the pilot, workshops with faculty and students were organised led by external experts on workplace-based assessment, programmatic assessment, and change management. Aim of the workshops was to find consensus about the building blocks of the assessment programme (e.g. goals, instruments). Subsequently, all participating faculty-members and students received a hands-on training in providing and seeking feedback on the clinical workplace and received information about the design and goals of the assessment programme.

## Methods

### Questionnaire and group interviews

To evaluate the assessment programme, we collected quantitative ratings (five-point Likert scale) on items from the quality assurance questionnaire administered after each clinical rotation, relating to feedback, supervision, assessment, and learning activities. The fifteen items related to these issues were completed on a five-point likert scale (1 = fully disagree and 5 = fully agree). A score of >3.5 was assumed to indicate attainment of the objectives of the assessment programme. These quantitative data provided starting points for further inquiry during group interviews. The latter are generally considered to be a suitable method for encouraging open discussion of views to yield in-depth information [16]. The interviews were structured around the four core elements of the programmatic approach described by Van der Vleuten et al. [13]: learning activities, assessment activities, supporting activities, and evaluation activities. The interviewees were asked to consider elements of the programmatic design that they thought stimulated or impeded learning. Input for the group interviews was also provided by the minutes of the monthly meetings of the research team.

### Procedure and participants

In September 2010 85 students, entering their three years of clinical training, piloted the new assessment programme. From April 2011 until May 2012, these students voluntarily completed the quality assurance questionnaire. In May and June 2012, two student groups (S1 and S2) and one group of clinical supervisors (T1) were interviewed. The interviewees represented the three animal species tracks and had started the clinical programme in September 2010. All 85 students were invited to participate. After sending the invitational e-mail, 18 students volunteered to participate in the group interviews. The participating students were divided into two groups (eight and ten students). Also, fifteen clinical supervisors received an invitational e-mail to join a group interview. The first eight supervisors volunteering to participate were invited. Each group interview lasted ninety minutes and was facilitated by a moderator (PvB). The interviews were audiotaped, transcribed verbatim, and participants were requested to comment on the accuracy of a summary of the interview. Three participants proposed minor additions.

### Analysis

Using SPSS version 20 we calculated mean scores for the quantitative data. The interview transcripts were analysed using software for qualitative data analysis (Atlas ti version 6.2.24). The first author (HGJB) wrote a preliminary descriptive summary of the findings and discussed it with the moderator until consensus was reached. The transcripts of the group interviews were coded resulting in a list of topics. Subsequently, these emerging topics were organized based on the research questions. The first author (HGJB) was responsible for coding the data and constructing the topics in lessons learned. The research team discussed the results until full agreement was reached.

### Confidentiality and ethical approval

The study was approved by the ethical review board of the Dutch Association for Medical Education (NVMO-ERB), and written informed consent was obtained from all interviewees. Participation was voluntary and participants were assured of confidentiality.

## Results

Between April 2011 and May 2012, 198 quality assurance questionnaires completed by 54 students (64% of total) were returned. The results for the selected items were analysed and discussed in the group interviews (Table 1). Of the eighteen participating students, sixteen were female and the mean age of the groups was 25.5 years (S1, range 23–32) and 25 years (S2, range 22–33). Of the eight participating clinical supervisors four were female and the mean age was 44.3 years (range 33–58). We present the results, with illustrative quotations, for each of the four research questions.

**Table 1:** Relevant items from the quality assurance questionnaire

**General course information (five-point Likert scale: 1 = fully disagree, 5 = fully agree)**		**Mean**	**SD**	**N**
1	My teachers take the initiative to evaluate my performance.	2.82	1.01	188
2	My teachers take the initiative to evaluate difficult situations in which I have been involved.	3.18	1.01	165
3	My teachers occasionally observe me when taking a history.	2.96	1.01	159
4	My teachers assess not only my veterinary expertise but also other competencies such as teamwork, organisational skills, and professional behaviour.	3.35	1.03	183
5	My teachers give regular feedback on my strengths and weaknesses.	3.42	0.91	183
6	It is useful to use a portfolio.	3.31	0.98	162
7	The portfolio gives me insight into my development as a professional.	3.02	0.95	161
8	The assessments in my portfolio are based on direct observation.	3.14	1.04	160
9	The information in my portfolio is based on observations of multiple tasks by multiple observers.	3.19	1.00	160
10	The mini-CEX-form allows me to document useful information.	3.45	0.59	60
11	The mini-CEX-form is easy to use.	3.08	0.95	61
12	At the start of a clinical rotation, arrangements are made about when to use a mini-CEX form for a direct observation.	2.21	0.89	61
13	I take the initiative for a mini-CEX.	4.24	0.63	59
14	Mini-CEXs enable me to identify my strengths and weaknesses.	3.56	0.63	57
15	It is easy for me to ask a clinical teacher to do a mini-CEX.	2.95	0.89	58

### Can data from multiple individual assessments be used to combine the formative and summative functions of assessment?

Students were expected to obtain feedback from mini-CEX and MSF. In the course of the programme students experienced more and more resistance to these instruments as they increasingly perceived the assessments as primarily summative rather than formative as intended by the programme designers. This made it difficult for students to attend to the formative aspects. Students felt the mini-CEX form emphasised the assessor role of the supervisor, especially due to the overall numerical rating and the fact that the scores on the competency domains were recorded in the portfolio, which was also used for summative assessment.

“Because my clinical supervisor has to fill in an assessment form, I cannot make a distinction between his or her role as assessor and coach. Therefore, a mini-CEX is not formative in my opinion.” (S2)

Despite their increasing reluctance to use the WBA instruments, students indicated a need for meaningful formative feedback and acknowledged the importance of documenting feedback. They experienced peer feedback as truly formative and used it to monitor their competency development.

“While doing clinical work I learn a lot from senior students. … they observe my performance and give valuable feedback indicating how I can improve.” (S2)

The value of peer feedback was recognised by clinical supervisors too:

“Within the ICU (Intensive Care Unit) a senior student and a junior student have to work as a team. I noticed that this responsibility has a positive effect on senior students, not only on their engagement with patient care but also on their willingness to give feedback to junior students.” (T1)

Clinical supervisors too experienced problems with the formative function of the assessment instruments. They expressed a desire to enter a pass/fail judgement on the assessment form and were unhappy that they had no influence over the weighing of individual assessments in the ultimate summative decision.

“In the previous assessment programme it was clear to me how my judgement of student performance influenced the summative score at the end of the clinical rotation. In the new programme I do not know if my feedback will be interpreted accurately and how it will affect the final mark.” (T1)

The findings raise doubts about the formative nature of individual assessments. While formative assessment implies assessment *for* learning, students perceived individual data points as primarily summative, i.e. as assessment *of* learning. This perception was due to assessments being recorded in the portfolio and used for summative decisions and it was reinforced by the generally low quality of the feedback.

### Can information from individual assessment data points be aggregated meaningfully?

The assessment programme comprised one intermediate and one final summative evaluation every year (Figure 2). The portfolio review committee (PRC) noticed that the monitoring of longitudinal competency development was impeded by the tendency of supervisors to give high marks and their difficulty in formulating high quality feedback (item 5, Table 1). Moreover, human professional judgement plays a crucial role in aggregating information from multiple, subjective, qualitative data sources for high-stakes decisions (promotion/remediation), and PRC members felt they were not ready for this role and found it hard to judge student portfolios against the benchmark of competence at graduation level. Another problem noticed by students and supervisors was that evaluation activities (items 7 and 9, Table 1) were not well aligned with learning and assessment activities. This was mainly due to poor alignment of students’ individualised training programmes with the rigid scheduling of evaluations.

“The portfolio review committee experienced difficulty comparing student portfolios because students’ training programmes are individualised while the intermediate and final evaluations are scheduled annually. Consequently, students have different amounts of data points in their portfolios, and a lot of variation can be seen between the evidence compiled.” (From minutes meeting portfolio review committee)

The evaluation activities depended heavily on the quality and expertise of judges. These summative evaluation are based on information derived from multiple individual formative assessments containing meaningful and information-rich feedback. Formative assessment tasks are thus similar to diagnostic expertise tasks, making specific demands on teachers skills and consequently on teacher training programmes. Difficulties in visualising students’ competency development were linked to ratings being generally above students’ true performance levels, poor qualitative feedback, and the difficulty of collecting feedback on all the required competencies. Clinical supervisors appeared to need more extensive training in the use of the WBA instruments, while the PRC called for on the job training, constant feedback, and supervision.

### Can assessment drive desirable learning?

Students indicated that it was difficult for them to monitor their competency development (items 5, 7, Table 1) due to shortcomings in the use of the WBA instruments. Initially, clinical supervisors had to get used to the new instruments, but apart from this temporary problem there was a general feeling among students and the PRC that feedback from clinical supervisors was not sufficiently specific and meaningful and focused on what went well rather than on enhancing student learning.

“The feedback I received on my performance was not specific enough, because the clinical supervisor did not observe my performance at all, he could only make some general comments.” (S1)

Both qualitative and quantitative information (items 1, 2, 3, 8, 12, 13, 15, Table 1) indicated that it was difficult for students to take responsibility for their own learning process, partly due to students’ reluctance to add to their supervisors’ workload by asking for feedback and partly due to supervisors’ busy schedules:

“During patient rounds there is no time to write down feedback in students’ digital portfolios. I give oral feedback, which they should record in their portfolio.” (T1)

It seems that effective use of WBA instruments to drive learning and provide meaningful feedback is conditional on proper feedback and assessment training. Students need feedback seeking skills, while supervisors need skills to provide appropriate qualitative feedback.

### How can reflective and self-directed learning activities be promoted?

Although six peer group sessions every year enabled students to discuss their learning goals, students indicated a preference for sessions with an individual coach or mentor, preferably the same one throughout their clinical training, who was familiar with their individual competency development.

“I feel that the evidence I am collecting in my portfolio is not visible to anyone. At this stage of my training I feel the need for more personal guidance from someone who really has insight into my competency development and can advise me. This should be my mentor.” (S2)

Reflective behaviour was not sufficiently promoted by the peer group meetings, which were considered to be ineffective in connecting supporting and evaluation activities with specific learning and assessment activities. It appears to be important to scaffold self-directed learning by offering students social interaction and external direction from a personal mentor.

## Discussion

The evaluations indicate that designing and implementing a competency-based assessment programme poses quite a challenge and demands intensive preparation and perseverance. The theoretical principles provided useful guidelines, and evaluating the programme and formulating lessons learned were vital steps towards improving the programme. The mixed composition of the research team (containing both clinical supervisors and educational researchers) was a key factor during the development and implementation phase. The clinical staff members on the research team played an invaluable role in facilitating the transfer of the assessment programme on paper to its implementation in practice. We will discuss the answers to each of the research questions.

### Can data from multiple individual assessments be used to combine the formative and summative functions of assessment?

The evaluation data provided no conclusive answer to the question if formative and summative functions of assessment can be combined in multiple assessment data points. Despite general acceptance of the usefulness of WBA instruments for formative assessment, their value for summative purposes is disputed [17,18]. The definition of *formative* assessment as used in the FVMU assessment programme proved to be misleading. The fact that all data points ultimately contributed to the final summative decisions caused students to perceive all individual assessments as summative rather than formative. In the eyes of the students, the final summative judgement was merely postponed until after the data points from the assessments were aggregated. The mismatch between the intended purpose of individual assessments and students’ perceptions of its role may partly be explained by students’ and teachers’ insufficient preparation for and instruction about the new programme. The programme designers may have underestimated the fundamental importance of faculty development and student training. Furthermore, it seems that the criteria for the final assessment could have been explained more clearly: which performance standards were used, how data were aggregated, how the final mark was determined, which remediation programmes were possible, and which purposes were served by the assessment programme. If students and clinical supervisors would have interpreted the value of individual low-stakes assessments in the same way students may have been better able to focus on the potential learning value of WBAs rather than on their summative consequences.

### Can information from individual assessment data points be aggregated meaningfully?

In the FVMU assessment programme a competency framework is used to aggregate information from individual data points of similar content [12,15]. Since what a test or item assesses is not determined by its format but by its content [19] and considering that assessments should not be trivialised in the pursuit of objectivity (e.g. by designing scoring rubrics for portfolios [20]) it seems of the utmost importance that in programmes of assessment subjective elements should be optimised by the sampling procedure and by combining information from various sources in a qualitatively meaningful manner [7]. Inevitably, this involves human judgement implying that the quality and expertise of judges are crucial for the quality of assessment [21,22]. This has important implications for teacher training. A single briefing, workshop, or training session does not suffice for assessors to reach the required level of expertise. On the job training, constant feedback, and supervision are needed [12]. This is in line with the findings from this evaluation, and we consequently redesigned the programme by including biweekly PCW meetings for training purposes and to exchange experiences.

### Can assessment drive desirable learning?

In their theoretical model Van der Vleuten et al. defined learning and assessment activities as two separate entities whose boundaries are blurred [13]. Assessment activities are part of the learning programme [23], but can they drive desirable learning? During the clinical clerkships students encountered many and varied learning activities (physical examination, history taking, ward rounds) each offering potential assessment opportunities. According to Prideaux, assessment and learning should be aligned to achieve the same goals and outcomes [24]. This is congruent with the principle that all assessment activities, and as a consequence all learning activities, should be maximally meaningful to learning. This is consistent with the conceptual shift from assessment *of* learning to assessment *for* learning [25], and further still to assessment *as* learning. Previous studies have shown that trainees indicated a need for structure and guidance in the transition from novice to the level of being competent. A programme of assessment containing instruments structured to facilitate this process, could support learning and monitor progression at higher levels of professional development [7,8]. The FVMU assessment programme, however, appears to have failed in creating an environment that gives full reign to assessment *for* learning. Feedback appears to have been the main stumbling block. Perceiving all WBAs as summative and a burden to supervisors, students were reluctant to ask for assessment with feedback, while supervisors claimed that time constraints impeded high quality feedback. This is in line with research reporting difficulties encountered while implementing tools to provide formative feedback [26,27]. Besides the poor quality of narrative feedback and the lack of direct observation, the administrative burden was mentioned as an explanation for trainees to perceive narrative formative feedback as not very useful [26,27]. For the coming years the main challenges will lie in creating a clinical environment that is intrinsically supportive of feedback, e.g. by simplifying documentation (e.g. user-friendly assessment instruments using mobile devices), feedback training for students and supervisors, and integrating WBA within the clinical organisation, as described in earlier research [28].

### How can reflective and self-directed learning activities be promoted?

From the literature we know that it can be quite a challenge to have students reflect upon feedback let alone use it to plan new learning tasks [29,30]. To address this problem Van der Vleuten and Schuwirth proposed a combination of scaffolding of self-directed learning with social interaction, leading to the peer group meetings in the programme [13]. Both students and supervisors acknowledged the value of peer feedback in teams of senior and junior students. Previous research also showed potential benefits of peer-assisted learning for both junior and senior students [31,32]. Ten Cate and Durning recognised the potential of peer-assisted learning during undergraduate clinical training, or “cognitive journeymanship”, and of incorporating valuable information from peer feedback (high-stakes assessment) [32]. The use of peer feedback is also in line with the notion that variety in instruments and sources is prerequisite for a complete picture of learner performance [10,33]. Recent research into students’ feedback-seeking behaviour during clinical clerkships showed that students sought information from different sources depending on a context-dependent assessment of the potential risks and benefits of feedback [34]. Apparently, when seeking feedback to achieve certain goals students strive to balance expected negative effects with potential benefits. We therefore propose to encourage teamwork during clinical rotations to encourage the use of feedback skills by students. Furthermore, students seemed to prefer social interaction and external direction by a personal mentor. This mentor could play an important role in guiding students to reflect on their past performance and in planning new learning goals. This is in line with literature stating that scaffolding of self-directed learning needs mentoring [29].

## Conclusions

To conclude, we would like to stress that putting assessment theory into practice by creating an environment that is conducive to assessment *for* learning requires careful attention to the implementation process. More specifically, it is essential to provide assessment and feedback training for students and supervisors, incorporate WBA within the organisation of clinics and wards, and design user-friendly WBA instruments. Quality feedback from clinical supervisors seems to be at the heart of the assessment process. In the FVMU assessment programme we found tension between the learning aspect of assessment and its contribution to high-stakes decisions. The difficulty of combining these two functions clearly needs further study The issue of whether or not assessment forms should require quantitative ratings seems another topic for further consideration. The need to give a quantitative mark may have offered an excuse for refraining from narrative qualitative feedback. Other strategies for enhancing the quality of feedback that should be investigated are the use of modern technology (e.g. handheld devices to record feedback, voice recorders) or the use of scoring rubrics.

### Future research

The findings of this study reveal a plethora of opportunities for further research. Besides the topics proposed by Van der Vleuten et al. [13] we would be especially interested in determining under which circumstances formative and summative assessment can be combined and on students’ and supervisors’ views regarding this issue. The influence of peer feedback on student learning and its potential role in an assessment programme deserve further study as well. Studies might also pursue promising developments in digital assessment tools to facilitate the capturing of feedback, enhance the quality of feedback, and reduce assessor workload.

## Competing interest

The authors declare that they have no competing interests.

## Authors’ contributions

All authors contributed substantially to the conception and design of the study. HGJB, RPF, NJR, LFHT, HB and JCMH were responsible for facilitating the implementation in practice. HGJB, PvB and DADCJ acquired all data and took responsibility for the analysis and interpretation of the data in collaboration with PWT and CPMvdV. HGJB wrote the first draft of the manuscript in collaboration with PWT and DADCJ. All authors read and approved the final manuscript.

## Pre-publication history

The pre-publication history for this paper can be accessed here:

http://www.biomedcentral.com/1472-6920/13/123/prepub
